# Digital health literacy among people with bipolar disorder in Germany – a cross-sectional survey

**DOI:** 10.3389/fpsyt.2025.1651938

**Published:** 2025-10-14

**Authors:** Linda Kokwaro, Helena Krüger, Dennis Stratmann, Daniel Schulze, Daniel Fürstenau, Surjo R. Soekadar, Sonia Lech, Stefanie Schreiter

**Affiliations:** ^1^ Department of Psychiatry and Neurosciences, and Berlin Institute of Health, Charité - Universitätsmedizin Berlin, Corporate Member of Freie Universität Berlin, Humboldt-Universität zu Berlin, Berlin, Germany; ^2^ Einstein Center for Neuroscience, Charité - Universitätsmedizin Berlin, Corporate Member of Freie Universität Berlin, Humboldt-Universität Berlin, and Berlin Institute of Health, Berlin, Germany; ^3^ International Psychoanalytic University, Berlin, Germany; ^4^ Institute of Biometry and Clinical Epidemiology, Charité - Universitätsmedizin Berlin, Berlin, Germany; ^5^ School of Business & Economics, Freie Universität Berlin, Berlin, Germany; ^6^ Institute of Medical Informatics, Charité - Universitätsmedizin Berlin, Corporate Member of Freie Universität Berlin, Humboldt-Universität Berlin, Berlin, Germany

**Keywords:** bipolar disorder, digital health literacy, structural, situational, technical access factors

## Abstract

**Background:**

This study assesses digital health literacy (DHL) among individuals with bipolar disorder (BD) in Germany, employing Sørensen’s *Integrated Model of Health Literacy (SIMHL).* According to SIMHL, health literacy is a dynamic construct influenced by demographic, structural, and situational factors. With increased adoption of digital health resources, often overlooked are the skills required to use these tools. This study aims to bridge this gap by examining overall patterns of DHL among individuals with BD and employing SIMHL to investigate DHL the role of associated structural, situational/clinical, and technical access factors.

**Methods:**

A cross-sectional online survey was conducted comprising 212 individuals with self-reported BD. DHL was assessed using the HL-DIGI instrument in addition to SIMHL factors including: structural (gender, employment status), situational (manic and depressive symptomatology), and technology use factors (use of health-related websites). Structural Equation Modeling (SEM) was used to model DHL as a latent variable.

**Results:**

DHL followed a bimodal distribution, with participants clustering at either low (33.5%) or high (31.6%) levels. Evaluating reliability of online health information and using it to solve health-related tasks proved to be the most challenging. SEM demonstrated overall adequate model fit with higher DHL significantly associated with male gender, being employed and use of health-related websites.

**Discussion:**

In line with SIMHL, DHL in individuals with BD is shaped by structural and access-related factors rather than illness severity. Gender, employment status, and engagement with health-related websites emerged as key correlates.

**Conclusion:**

DHL in BD is not uniformly lower compared to the general population, underlining the feasibility of digital interventions as a promising pillar of care. At the same time, substantial heterogeneity highlights the need for routine DHL screening and tailored support for subgroups with lower skills. Future interventions and studies should systematically stratify by DHL level and target higher-order skills such as evaluating reliability and decision-making, in order to ensure equitable access and maximize the benefits of digital mental health care.

## Introduction

Digital Health Literacy (DHL) is increasingly recognized as a prerequisite for equitable participation in healthcare (1). The World Health Organization (WHO) conceptualizes DHL as, “The ability to search, find, understand and evaluate health information from electronic resources and to use the knowledge gained to solve health-related problems” (2). Often used interchangeably with eHealth literacy, DHL extends beyond technical skills to include evaluative, participatory and privacy-related competencies that enable meaningful engagement with digital health services (2, 3).

Despite its importance, population-level studies consistently show DHL to be limited. In Germany, the Second Health Literacy Survey Germany (HLS-GER2) reported that 75.8% of the general population had low DHL, substantially higher than the 58.8% with low general (non-digital) health literacy (3). This decline over time has been linked to the growing volume of digital information, the spread of misinformation and greater demands on information processing (4, 5). DHL also follows a social gradient, with lower scores observed among older adults, individuals with low socioeconomic status, low levels of education and functional literacy and those with multiple chronic illnesses (6). A representative study by a German statutory health insurance provider (AOK) similarly found that 52.4% of respondents reported limited DHL, particularly in evaluating reliability and relevance of health information (7). At the policy level, a WHO European region assessment across 53 member states, revealed that only 52% had formal DHL or digital inclusion strategies (8). A recent scoping review on DHL further emphasized that misinformation and digital complexity are exacerbating inequities (9).

People diagnosed with bipolar disorder (BD) face distinct barriers to acquiring and applying health information online, including illness complexity, comorbidities, and cognitive difficulties during acute phases (10). To date the only large scale study to directly assess DHL in individuals with BD is the international web-based survey by Morton et al., which found that BD participants (n = 919) reported relatively high self-rated DHL levels, comparable to, or even exceeding previous assessments in the general population samples (10, 11). Furthermore, factors such as older age, postgraduate education and current use of BD related self-management apps were found to be associated with high DHL levels (10). However, this study uses the eHealth Literacy Scale (eHEALS) instrument, which measures perceived rather than actual DHL, and did not examine illness-specific factors or broader digital engagement (12).

Research in other Serious Mental Illness (SMI) populations including psychosis-spectrum disorders highlights the role of gender, prior internet knowledge, and regional infrastructure, with large variations across countries even within the same diagnostic group (13, 14). Most such studies were conducted prior to or during the early stages of the COVID-19 pandemic, when digital reliance increased sharply and health misinformation proliferated (15–17). During the course of the pandemic, factors such as stringent access to in person care, unstable housing, social stigma, isolation and economic strain have been posited to influence the general care of BD patients (18). Furthermore, restrictive measures during the pandemic could have motivated people to engage more in digital technologies. Additionally, there has been an overabundance of health information online and an upscale of digital mental health interventions such as monitoring apps for BD, which could further affect current DHL scores (19–24). These shifts may have fundamentally altered DHL in SMI populations.

### Aim of the present study

The key objective of the study was to investigate DHL among individuals with BD using *Sørensen’s Integrated Model of Health Literacy (SIMHL)* which incorporates structural, situational, and technological determinants (25). Specifically, this study aims (i) to examine overall patterns of DHL and (ii) to investigate associations between DHL and a range of theoretically informed covariates using a structural model grounded in SIMHL. Based on SIMHL, the social gradient reported to be associated with DHL (age, gender, education), use of digital health resources (prior internet knowledge, self-management apps), known clinical findings on the burden of disease (severity of symptoms, chronicity of illness) among people with BD, we formulated the following hypotheses: H1 (Structural Factors): *Lower DHL is associated with male gender, lower educational attainment, and advanced age*. H2 (Situational/Clinical Factors): *Lower internal locus of control as a proxy for self-efficacy, recent affective episode, earlier symptom onset, later diagnosis and higher manic and depressive symptom severity, are negatively associated with DHL*. H3 (Technological Access): *Technological access, operationalized as use of digital health resources, including health-related websites, social media and online forums, smartphone apps, wearable devices, and remote communication with healthcare providers, is associated with higher DHL*.

## Methods

### Study design and setting

We conducted a nationwide, anonymous open online survey among individuals with BD between August 2023 and January 2024 in Germany. Ethical approval for this study was obtained from the Charité - Universitätsmedizin Berlin ethics committee (No. EA1/218/22). The survey was anonymous, allowing for increased participation, more open and honest responses and reduced social desirability bias, while enhancing privacy and confidentiality. To expand the reach and diversify the range of respondents, we engaged people who are part of (online) BD self-help/support groups during the recruitment phase to create dissemination activities and materials. Subsequently, we leveraged already established connections through the Department of Psychiatry and Neurosciences at Charité with links to associations like the German Society of Bipolar Disorder and Bipolaris e.V., Pinel initiative (only organization that offers Ex-In training for people with lived psychiatric experience).

### Participants

The quantitative phase of the study aimed to recruit a sample of 250 participants using convenience sampling. Inclusion criteria required participants to be at least 18 years of age and to have a self-reported diagnosis of BD. Prior to participation, respondents were provided with detailed information regarding the estimated duration of the survey, data management procedures (including storage location and retention period), and the aims of the study. Informed consent was obtained electronically via a consent button, which outlined the general terms and conditions of the study. Diagnostic data were collected through self-report, and participants were further asked to indicate the type of BD diagnosis received as well as the type of professional that issued the diagnosis.

### Survey development

The survey was developed through a collaborative process involving thirteen individuals, including persons who lived with BD, peer support specialists, and caregivers, who participated in a two-day co-creation workshop. Eligibility criteria for participation in the workshop were as follows: a minimum age of 18 years, a confirmed diagnosis of BD (ICD-10: F31) for people with lived BD experience, the capacity to provide informed consent, a euthymic state sustained for at least two weeks or the presence of mild to moderate depressive symptoms (as determined by validated cut-off scores on the QIDS-SR and ASRM), and sufficient proficiency in the German language. Exclusion criteria encompassed acute suicidality, the presence of a current severe affective episode or (hypo)manic state (as defined by clinical thresholds on the QIDS-SR and ASRM), any organic brain disorder, cognitive impairments, and an inability to comprehend the study protocol or associated risks. Additionally, physical illnesses of a nature or severity likely to interfere with the study procedures or influence study outcomes were grounds for exclusion.

Participants, hereafter referred to as co-researchers, were provided with training on the current state of research in the field, the application of quantitative survey methodologies, and the formulation of inclusive and appropriately structured survey items. Techniques such as the use of Likert scales were introduced to ensure that the survey was both engaging and accessible to potential respondents. The co-researchers played a central role in identifying and prioritizing core survey items and in designing adaptive, logic-based question flows aimed at optimizing data completeness while maintaining participant comfort. Usability and technical testing of the survey were conducted by the co-researchers prior to deployment, and their feedback was systematically integrated into the final version of the instrument.

### Variables and instruments

*Digital Health literacy:* DHL was assessed using a total of 10 items that are part of the HLS_19_-DIGI (Health Literacy – Digital) questionnaire, which is used to measure general digital health literacy und use of digital health resources within adult populations. The DHL part of the instrument is henceforth referred to as the HL-DIGI instrument, which included 8 items on DHL and 2 items to measure interaction with digital devices (26). An example item was, “*When you search for health information online, how easy or difficult is it for you/is it very easy, easy, difficult or very difficult for you to use the right words or search terms to find the information you need (online)?”* The HL-DIGI instrument captures aspects such as the ability to use search terms, understand information, check websites, find the right information, search for information, trust information, solve health-related tasks/problems and evaluate information, compose a message and express an opinion. Each item was rated on a 5-point Likert scale (“very difficult” to “very easy”), with scores rescaled to range from 0 to 100. Participants were then categorized into DHL levels based on cut-off scores adapted from the HLS-EU (Health Literacy European) framework: Excellent DHL: >83.33% (≥10 out of 12 items rated “easy” or “very easy”), 2) Satisfactory DHL: >66.67% and ≤83.33% (8–10 items rated “easy”/”very easy”), 3)Problematic DHL: >50% and ≤66.67% (6–8 items rated “easy”/”very easy”), and 4) Inadequate DHL: ≤50% (≤6 items rated “easy”/”very easy”). Additionally, item-level analysis was conducted to explore perceived task difficulty. The proportion of participants who rated each task as “difficult” or “very difficult” was calculated and visualized to highlight areas of challenge across different domains of DHL.

*Technology Access*: Technology access, operationalized as the utilization of digital health resources, was assessed using the 6 item HL-DIGI-DD instrument derived from the HLS_19_-DIGI questionnaire (26). The subcategories assessed include i) the use of health-related websites, ii) social media and online forums for health-related content, iii) digital health devices, e.g., smartwatches, iv) smartphone health apps, v) digital interaction with healthcare providers and vi) the use of other general digital health resources. An example of a question was, “*On how many days in a typical week do you use health-related websites to find out information about health issues?”*. A 7-point Likert scale ranging from less than once a week to having no experience at all was used to assess the responses.

*Locus of control:* Locus of Control was measured using the Internal–External Locus of Control Short Scale-4 (IE-4), a validated 4-item instrument that captures generalized control beliefs along two dimensions: internal and external (27). An example item from the IE-4 scale was Internal LOC item (Item 1): *“If I work hard, I will succeed.”* An example of an External LOC item (Item 2, reverse-coded) was: *“Whether or not I am successful in life depends mostly on luck.* “The items reflect the belief that outcomes are driven either by one’s own actions (internal) or by external forces beyond one’s control (external). Items were rated on a 5-point Likert scale ranging from 1 (“does not apply at all”) to 5 (“fully applies”). To calculate the score, items 2 and 4, which assess external control beliefs, were reverse coded to ensure alignment in the directionality of all items. A total locus of control score was computed as the mean of all four items, with higher scores reflecting a stronger internal locus of control. Additionally, two subscale scores were derived: Internal locus of control score: Mean of items 1 and 3 (internal beliefs) and 2) External locus of control score: Mean of reverse-coded items 2 and 4 (external beliefs, reverse-coded to reflect higher internality). These subscales allowed for more granular analysis of the relationships between internal and external control orientations and digital health literacy (27). For the SEM, only the internal locus of control subscale was included as an observed variable. This decision was informed by theoretical considerations linking internal locus of control to self-efficacy, a key psychological construct influencing individuals’ ability to actively seek, evaluate, and use health information, core components of DHL. In contrast, external locus of control is associated with perceived helplessness or passivity, which are less likely to promote active engagement with digital health resources. Thus, internal control beliefs were treated as a proxy for self-efficacy in predicting DHL outcomes.

*Bipolar symptomatology:* Current depressive and manic symptoms were assessed using the Quick Inventory of Depressive Symptomatology (QIDS-SR) and Altman Self-Rating Mania Scale (ASRM) respectively (28, 29). The QIDS-SR measures severity of depressive symptoms with scores ranging from 0-27, interpreted as follows: 0-5 (no depression), 6-10 (mild depression), 11-15 (moderate depression), 16-20 (severe depression), and 21-27 (very severe depression). The ASRM assesses manic symptoms on a scale of 0-20, with scores interpreted as 0-5 (no significant manic symptoms), 6-9 (mild to moderate manic symptoms), and ≥10 (high likelihood of mania).

### Data collection

The survey was distributed nationwide in Germany primarily through online and offline means. For online channels, social media, online forums and patient support groups were used. For offline channels, the survey was disseminated to university and outpatient clinics and local patient support groups with additional help of co-researchers. The survey was published the 23/08/23 and was live until 07/01/24. Data was collected via Redcap.

### Data analyses

#### Descriptive statistics

All statistical analyses were conducted using R (v4.3.2). Means and standard deviations were calculated for continuous variables; frequencies and proportions were used for categorical variables. Normality of continuous data was assessed visually (histograms and Q-Q plots) and statistically (Shapiro-Wilk test). Where assumptions of normality were not met, non-parametric tests (e.g., Mann-Whitney U) were used in place of t-tests. Self-reported age of symptom onset values were screened for both statistical and clinical plausibility. Outliers were identified using Tukey’s interquartile range (IQR) method. Sensitivity analyses were performed restricting the age of symptom onset to 14–50 years, a range encompassing typical early-, mid-, and late-onset BD while excluding extreme values prone to recall error or atypical etiologies. This range is consistent with pooled epidemiological data showing trimodal peaks at approximately 17, 26, and 42 years (30). All primary analyses were conducted on the full dataset, with sensitivity analyses repeated on the restricted 14- 50-year subset to evaluate stability of results.

Categorical group differences were assessed using Chi-square tests. Missing or out-of-range values were explicitly coded as NA and handled according to the requirements of each analysis. For descriptive statistics, missing values were excluded using na.rm = TRUE. For confirmatory factor analyses (CFA) and structural equation modeling (SEM) conducted using the lavaan package in R, different strategies were applied depending on the estimator. For models using Weighted Least Squares Mean and Variance adjusted (WLSMV), listwise deletion (missing = “listwise”) was employed to ensure consistent case inclusion across ordinal indicators. Ordered = TRUE argument was additionally used for ordinal indicators in WLSMV estimation and that only complete cases were included in those analyses. For models estimated with Maximum Likelihood with Robust standard errors (MLR), the default full information maximum likelihood (FIML) approach was used to account for missing data under the assumption of missing at random (MAR).

Structural Equation Modeling (SEM) provided a robust framework for examining complex, interrelated variables influencing DHL. Unlike traditional regression, SEM accommodates latent variables, controls for measurement error, and enables simultaneous estimation of direct and indirect effects. This approach is particularly important for modeling multifaceted constructs like DHL, which encompass cognitive, technical, and health-related domains. To examine factors associated with DHL, a confirmatory factor analysis (CFA) was conducted: a latent variable model was estimated using the Weighted Least Squares Mean and Variance adjusted (WLSMV) estimator, appropriate for ordinal indicators. The latent DHL construct was defined by the 10 HL-DIGI items. Given that the focus of the study was on SEM models and psychometric properties of the HL-DIGI scale, we determined the number of participants by rules of thumb for planning factor analyses. Frequently, these state that 10 to 20 participants per item (31). Having 10 items, we thus aimed for 200 participants. The latent construct of DHL was regressed on the following observed variables: Sociodemographic variables: age, gender (1 = male, 2 = female), educational level, Clinical variables: age of symptom onset, recency of last affective episode (in months), current manic symptom severity (ASRM score), depressive symptomatology (QIDS-SR score), self-efficacy score and Technology use variables: Use of digital health resources, including health-related websites, social media and online forums, smartphone apps, wearable devices, and remote communication with healthcare providers. Model fit was evaluated using conventional SEM indices: the Root Mean Square Error of Approximation (RMSEA), Comparative Fit Index (CFI), Tucker-Lewis Index (TLI), and Standardized Root Mean Square Residual (SRMR). Standardized beta coefficients and significance values were interpreted to evaluate support for each hypothesis.

## Results

### Baseline characteristics

A total of N = 321 individuals clicked on the link to the online survey. Of these, N = 109 participants (34%) were excluded because they either clicked through the survey quickly without answering questions or spent less than 5 minutes on the survey. The final analytic sample thus consisted of N = 212 participants. To assess potential bias due to exclusion, we compared available demographic and partial response data (e.g. age, gender, partially completed target variables) between excluded and included cases. These descriptive comparisons did not reveal meaningful differences, suggesting that data were likely Missing Completely at Random (MCAR). Therefore, the final sample (*N* = 212) can be considered representative of the initial pool of respondents for the retained variables. The final sample had a mean age of 45.5 years (SD = 12.5; range: 18-78), with 65.3% identifying as female, 29.1% as male, and 5.6% as diverse. Regarding relationship status, 39.9% were single; 39.4% were living alone and 43.7% with family members. Educational attainment was relatively high, with 80.2% having earned a higher education entrance qualification. Regarding employment status, the majority were either employed (40.1%) or retired (37.3%). Among those employed, 51.8% were working part-time. Of the retired individuals, 83.5% were in early retirement due to reduced earning capacity. Clinical characteristics of the sample revealed a mean age of symptom onset at 23.13 years (SD = 11.3; range: 5-62), while the average age at diagnosis was 35.38 years (SD = 10.7; range: 13-62), reflecting an average diagnostic delay of approximately 12 years. Current symptomatology: the mean ASRM score was 2.6 (SD = 3.6), with 15.6% of participants scoring 6 or higher, indicative of (hypo)manic symptoms. The mean QIDS-SR score was 10.9 (SD = 5.3), with 14.6% scoring 16 or above, consistent with mild to moderate depressive symptomatology. Additionally, respondents reported slightly higher internal (M = 2.96, SD = 0.49; range: 1-5) than external locus of control scores (M = 2.85, SD = 0.72; range: 1-5). The two subscales were moderately negatively correlated (r = -0.25), indicating that individuals with stronger beliefs in their own agency tended to be less likely to attribute outcomes to external factors. However, the inverse was not absolute, suggesting nuanced beliefs about control among this population. Comprehensive information on participant demographics and clinical features is presented in **Table 1**.

**Table 1:** Baseline characteristics of the sample.

Sociodemographic characteristics	*Total sample (N = 212)*, *n (%) or mean (SD, range)*
Age in years: mean	45.5 (12.5,18-78)
Gender	
Female	139 (65.6%)
Diverse	11 (5.6%)
Relationship status	
Single	85 (39.9%)
Married	72 (33.8%)
In a relationship	52 (24.4%)
Highest education level attained	
Lower secondary/no formal qualification	11 (5.7%)
Intermediate secondary certificate	27 (14.1%)
Higher education entrance qualification	154 (80.2%)
Employment Status	
Employed	85 (40.1%)
Retired	79 (37.3%)
Self-employed	12 (5.7%)
Receiving Unemployment Benefits I/II	14 (6.6%)
Student	11 (5.2%)
Clinical assessments	
Type of bipolar diagnosis	
Type 1	103 (48.6%)
Type 2	92 (43.4%)
Other	12 (5.7%)
Age of symptom onset: mean (*SD*, range)	23.1 (11.3, 5-62)
Age of diagnosis: mean (*SD*, range)	35.2 (10.7, 13-62)
QIDS-SR score: mean (*SD*, range)	10.9 (5.3, 0-27)
ASRM score: mean (*SD*, range)	2.6 (3.6, 0-20)
Internal Locus of Control: mean (*SD*, range)	3.0 (0.5, 1-5)
External Locus of Control: mean (*SD*, range)	2.8 (0.7, 1-5)
Total Locus of Control: mean (*SD*, range)	2.9 (0.4, 1-4)

Values are presented as mean (Standard Deviation (SD), range) for continuous variables and n (%) for categorical variables. QIDS-SR stands for Quick Inventory of Depressive Symptomatology and ASRM for Altman Self-Rating Mania Scale. Missing data were handled using listwise deletion. The number of missing values for each variable did not exceed 5% of the total sample.

### Findings on digital health literacy

**Figure 1:**
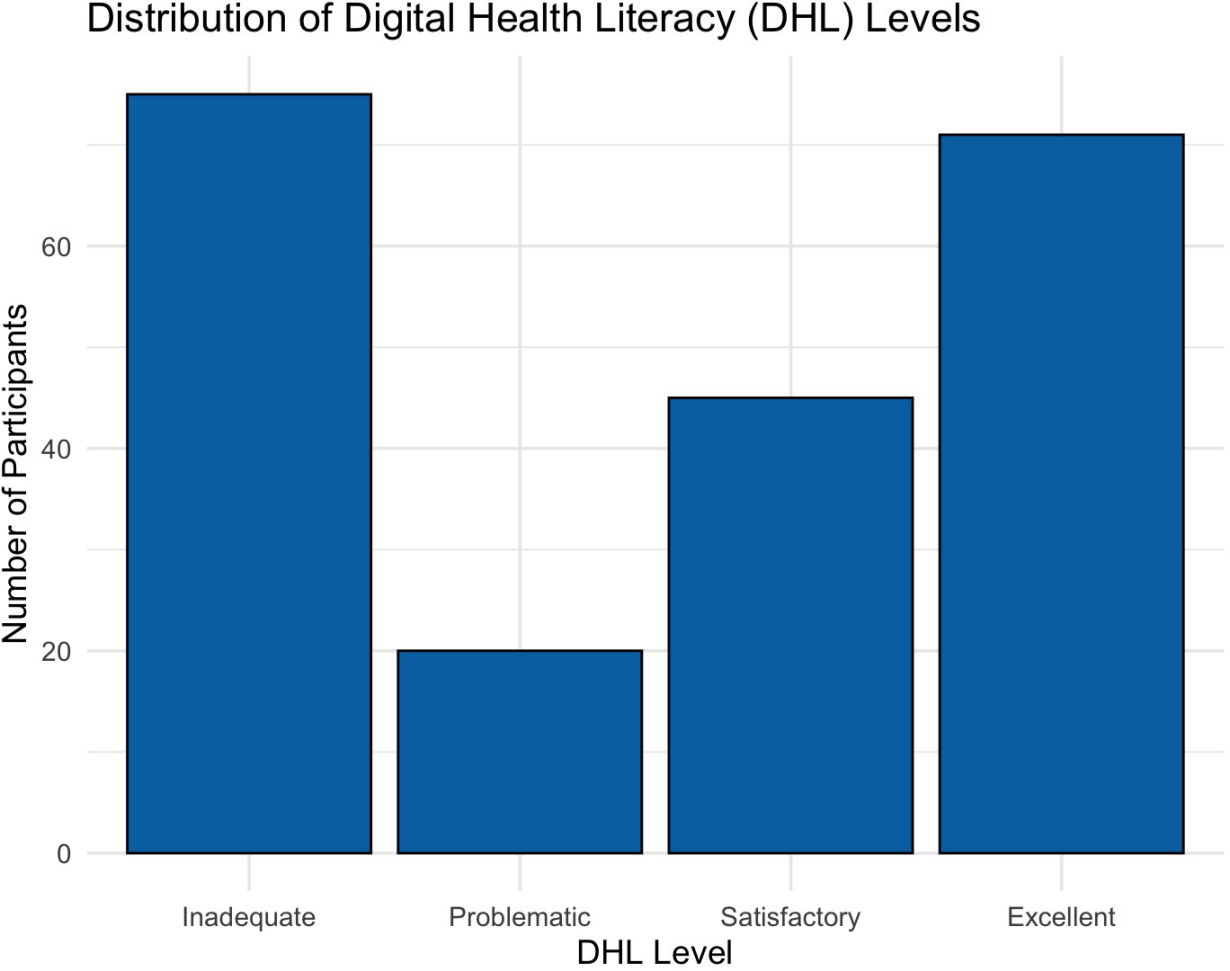
Distribution of Digital Health Literacy Levels.

*Note. Distribution of Digital Health Literacy (DHL) levels among participants. DHL scores were categorized into four levels: Inadequate* (0–50)*, Problematic (50.01–66.67), Satisfactory (66.68–83.33), and Excellent (83.34–100). The figure shows a bimodal distribution, with the largest proportions of participants falling into the Inadequate and Excellent categories.*

The distribution of DHL levels was bimodal, with the largest proportions of participants in the inadequate and excellent categories and fewer in the intermediate ranges. Specifically, 33.5% (n = 71) had inadequate DHL and 31.6% (n = 67) had excellent DHL, while 21. 2%(n = 45) and 9.4% (n = 20) were classified as satisfactory and problematic, respectively. **Figure 1** illustrates the distribution of participants across the four levels of DHL.

**Figure 2:**
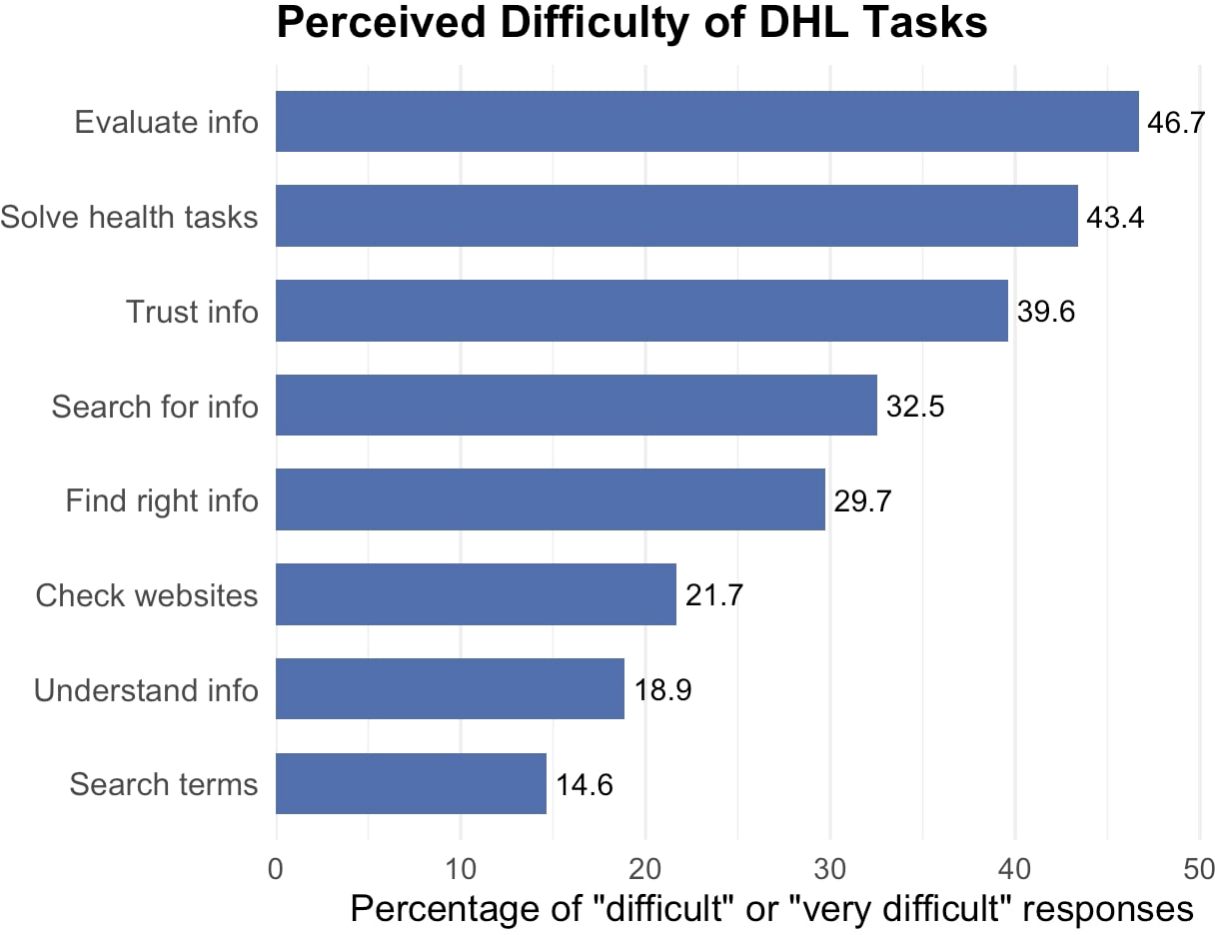
Perceived Difficulty of Digital Health Literacy (DHL) Tasks.

*Note*. Percentage of participants who rated each Digital Health Literacy (DHL) task as “difficult” or “very difficult”. The percentage of responses ranges from 14.6 to 46.6%. The tasks are ordered by increasing perceived difficulty as follows, ability to evaluate information, solve health related tasks/problems, trust information, search for information, find the right information, check websites, understand information and use search terms.

Regarding the perceived level of difficulty of DHL tasks, evaluating the reliability of digital health information (46.7%) and solving health-related tasks using online information (43.4%) were reported as the most difficult tasks respectively. These were followed by trusting online information (39.6%) and searching for the right kind of information (32.5%). In contrast, tasks such as choosing appropriate search terms (14.6%), understanding information (18.9%), and checking website credibility (21.7%) were perceived as the least difficult. **Figure 2** illustrates the perceived difficulty of various DHL tasks among participants, based on the percentage of respondents who rated each task as either “difficult” or “very difficult.

### Confirmatory factor analysis of the DHL scale

**Figure 3:**
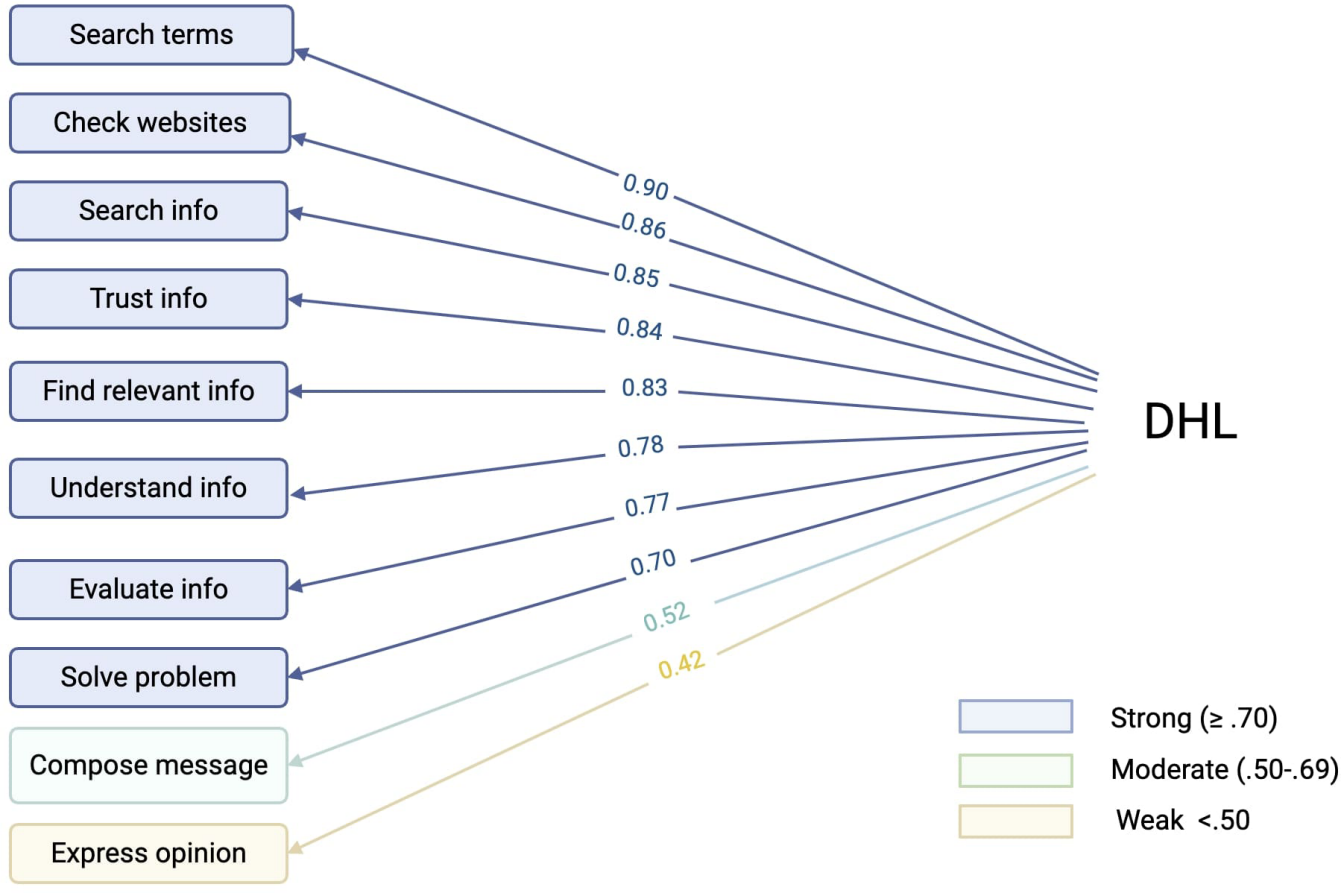
Path diagram of the confirmatory factor analysis (CFA) model assessing Digital Health Literacy (DHL).

*Note*. Path diagram of the confirmatory factor analysis (CFA) model assessing Digital Health Literacy (DHL). Standardized factor loadings (ranging from 0.42 to 0.90) are displayed along the arrows from the latent DHL factor to its 10 observed indicators.The observed indicators are as follows: trust the information, check websites, find the right information, express opinions, formulate messages, search for information, solve health related tasks/problems, use search terms, understand information and evaluate information. Strong indicators (blue): loadings ≥.70, Moderate indicators: loadings.50-.69 (green) and Weak indicators: loadings <.50 (yellow). Illustration created on Biorender.com.

A CFA was conducted to test the measurement model of DHL, specified as a single latent factor with 10 ordinal indicators (**Figure 3**). The model was estimated using the WLSMV estimator in lavaan (v0.6-19). The model showed acceptable fit based on CFI (0.988), TLI (0.984), and SRMR (.072), although RMSEA indicated poor fit (RMSEA = .128, 90% CI [.108,.149]). All factor loadings were statistically significant (p <.001). Standardized factor loadings ranged from 0.42 to 0.90, with the highest loadings for use of search terms (0.90) and checking the reliability of health-related websites (0.86), searching for health-related information online (0.85), and the lowest for expressing personal opinions on health issues online (0.42) as shown in **Figure 3**.

### Structural equation model: factors associated with DHL

**Figure 4:**
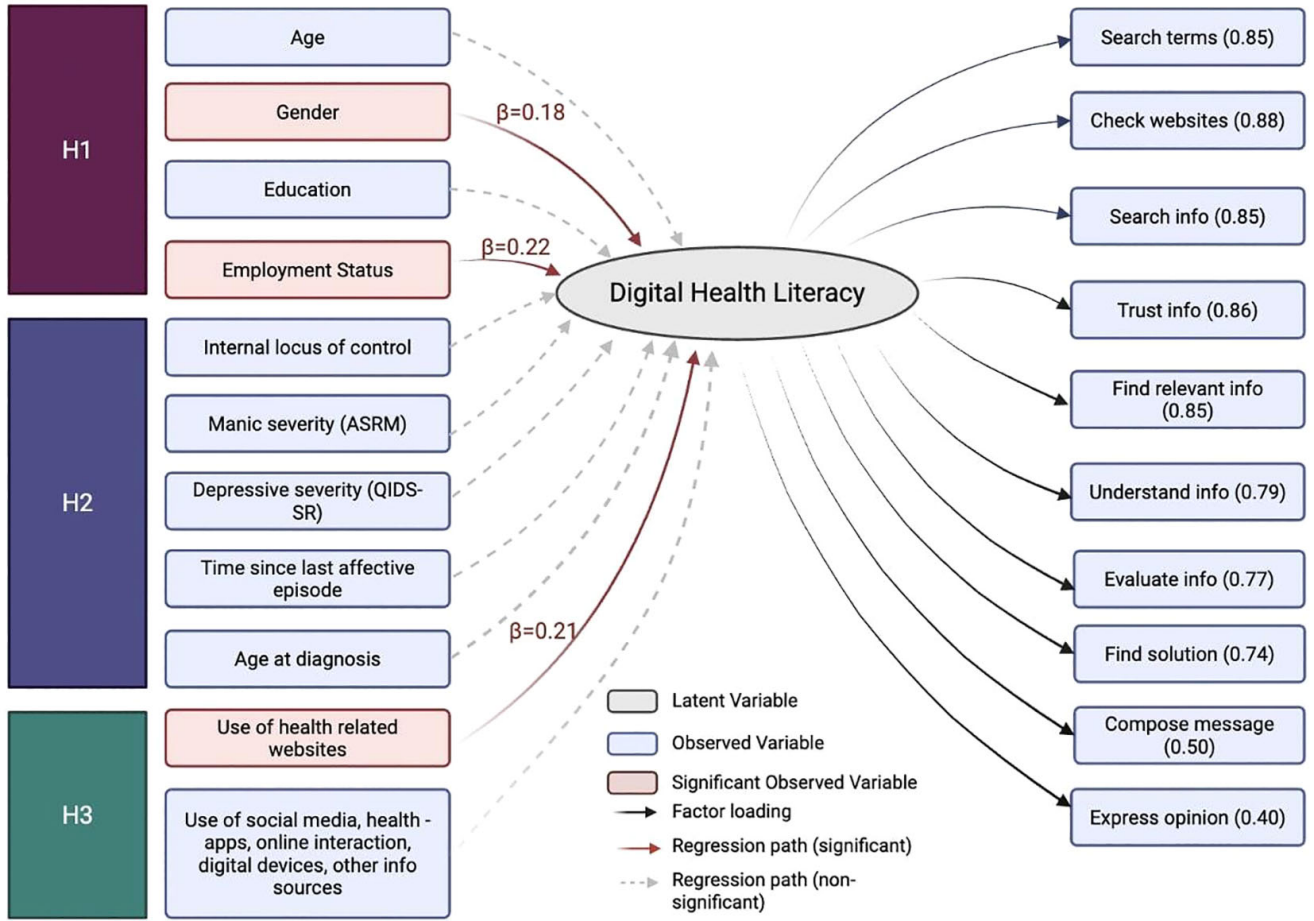
Structural Equation Model (SEM) of DHL and Associated Factors.

*Note*. Structural equation model estimating Digital Health Literacy (DHL) as a latent variable measured by ten observed indicators (right-hand side) and regressed on a set of theoretically informed covariates (left-hand side), including Hypothesis 1(H1): structural variables (age, gender, education and employment status), Hypothesis 2 (H2): situational/clinical variables (manic symptoms [ASRM], depressive symptoms [QIDS-SR], time since last affective episode, age at diagnosis, internal locus of control), and Hypothesis 3 (H3): technology access variables (use of health-related websites, social media use, digital devices, health app use, online interaction with providers and other digital health resources such as chat bots). Solid arrows represent statistically significant paths (*p* <.05), while dashed arrows indicate nonsignificant paths. Standardized factor loadings are displayed below each observed indicator. The model was estimated using WLSMV in lavaan, and factor loadings ranged from.40 to.88. Model fit indices indicated excellent fit: χ² (170) = 233.56, p = .001; CFI = .975; TLI = .993; RMSEA = .047 (90% CI [.031,.061]); SRMR = .064. Illustration created on Biorender.com.

In **Figure 4**, a final SEM model was estimated with multiple covariates: age, gender, education, employment status, internal locus of control, manic symptom severity (ASRM score), depressive symptom severity (QIDS-SR), time since last affective episode, age at diagnosis and use of various digital health resources (such as health-related websites and smartphone apps). The final model explained 18.2% of the variance in DHL (R² = .18). This model demonstrated adequate overall fit: χ² (170) = 233.56, p = .001; CFI = 0.98; TLI = 0.99; RMSEA = 0.05, 90% CI [0.03, 0.06]; SRMR = 0.06. Three variables were significantly associated with DHL: Gender (β = 0.18, *p* = .05), with males reporting higher DHL than females; employment status, (β = 0.22, *p* = .01) and use of health-related websites (β = 0.21, *p* = .01). Internal locus of control was not significantly associated with DHL (β = -0.10, p = .202), though the direction of association was negative. Other variables including age, education, digital health resource use items such as social media and health app use, symptom burden (QIDS-SR, ASRM), age at diagnosis, and time since last affective episode were not significantly associated with DHL. All factor loadings for the latent DHL construct were significant as shown in **Figure 4**.

## Discussion

This study provides insights on digital health literacy skills and associated factors among individuals with BD using a comprehensive SEM approach grounded in a framework on health literacy (Sørensen’s *Integrated Model of Health Literacy)*. Overall DHL levels were not consistently lower than in the general population but demonstrated a bimodal distribution. One subgroup demonstrated strong navigation and evaluation skills, while the other reported inadequate DHL, underlining the need for screening on DHL in clinical practice as well as taking this subgroup into account in the development of digital tools. Higher order tasks such as evaluating reliability and applying information to solve health-related problems, were perceived as most challenging, consistent with prior evidence that appraisal and application of information were the most demanding DHL components (7).

### Factors associated with DHL

The final SEM model identified structural factors including male gender and being employed (hypothesis 1) and the use of health-related websites (hypothesis 3) to be significantly associated with high DHL among individuals with BD. Clinical factors such as BD symptomatology (hypothesis 2) were not significantly associated with DHL. The association between male gender and higher DHL contrasts with our initial hypothesis and prior findings from general population studies and SMI cohorts, which often report higher DHL among females (13, 32). However, in a systematic review investigating sociodemographic determinants of DHL, no significant influence of sex was found, instead age had a significant negative influence on DHL and educational level, higher income, and social support appeared to have a positive influence (6). It could be that males may engage in more problem-focused online information-seeking behaviors and possibly demonstrate higher confidence in navigating digital environments (33). In addition to male gender, being employed was also linked to high DHL in our cohort. This finding aligns with the mentioned systematic review connecting DHL to higher income (6). In a study in the US examining predictors of self-management in digital mental health-services provided as a benefit of employment, baseline depressive symptom severity was linked to engaging in coaching or therapy online, even prior to having a crisis (34). Being employed may increase access to help-seeking pipelines, leading to higher functional stability and digital skill development. The use of health-related websites emerged as the only significant technology-associated factor associated with DHL, echoing previous studies which reported the use of the internet as the most consistent predictor of DHL in general (35–37).

The non-significant effect of education was unexpected given its well-established association with DHL (3). This may be due to the overall high educational attainment in our sample (80.2% held the higher education entrance qualification), potentially attenuating variability. Similarly, the lack of a significant association between Internal locus of control (LOC) and DHL might also reflect underlying nuances, where comparatively higher internal than external LoC scores suggest valuing personal agency, while still acknowledging external influences (38). This nuance aligns with findings that proactive digital engagement may require not only motivation but also openness to external guidance and trust in digital tools (39, 40).

### Clinical and policy implications

These findings highlight the need to tailor digital health interventions for individuals with lower DHL, particularly those with lower socioeconomic status e.g. not being employed or individuals less engaged with internet use. Moreover individuals with BD may face unique challenges due to cognitive vulnerabilities, fluctuating symptoms, and illness complexity (10, 41). Disparities in DHL threaten equitable adoption of digital (mental health) care (32) as many patients rely on peer or app-store recommendations (42, 43). However, a review on apps returned for the search term ‘bipolar’ revealed that most apps are irrelevant to BD and not following international guidelines (24, 44, 45). To support individuals with BD to navigate in this field, digital interventions in BD could routinely include an assessment of DHL, provide training and support for subgroups with lower DHL and embed DHL stratification as a design principle when developing, testing, or implementing digital interventions. Such strategies would ensure that digital innovations benefit a broader spectrum of patients rather than primarily those who are already digitally literate.

Currently, only few interventions target DHL in mental health populations and most of them focus narrowly on foundational digital skills (46, 47). There is a need for targeted, skills-based interventions that build evaluative and decision-making competencies and integrate these into platforms beyond apps, including telepsychiatry, wearables and peer-support networks. Identifying patients on the lower level of DHL in clinical practice such as individuals with a lower socioeconomic status e.g. being unemployed or individuals with a lower digital affinity (e g. rarely using health-related websites) has the potential to address increasing (digital) health equities. The use of the HL-DIGI instrument to assess DHL and the HL-DIGI-DD instrument to assess use of digital health resources e.g. before prescribing a digital therapeutic or using telepsychiatry with a patient could be one approach to identify individuals with the need to increase their DHL skills. The HL-DIGI-DD instrument should be updated to include present day common AI driven chat bots, given the rise in usage. In Germany, incorporating these DHL assessments at the general practitioner level could allow for identification of low proportion subgroups that could otherwise be lost in a referral-based health-care system. Establishing such routine assessments and stratification could be incorporated into national guidelines and funding schemes, ensuring that DHL is treated as a core determinant of equitable access to digital psychiatry. This is pertinent, since for instance the highest proportion of official digital therapeutics in Germany are approved in the field of mental disorders, making digital tools a growing pillar in care.

### Limitations

Several limitations should be acknowledged. The sample was skewed toward highly educated individuals who were recruited which may bias results towards digitally literate individuals, potentially inflating DHL estimates. Diagnoses and clinical characteristics were self-reported rather than confirmed through standardized interviews, which may introduce variability, although prior research suggests high validity for self-reported BD diagnoses (10). Although a subset of participants were excluded due to incomplete or rapid responses, comparisons showed no meaningful demographic or response differences, suggesting missingness was largely random. Still, reliance on listwise deletion in SEM reduced power and cannot fully exclude systematic bias. Future studies should consider multiple imputation or sensitivity analyses. The cross-sectional design of the study also precludes causal inference. Finally, while the HL-DIGI instrument offers advantages over perception based tools like eHEALS (48, 49), the assessment of DHL remains subjective and may not fully reflect functional literacy in real-world contexts (39). Future studies should aim to include more diverse samples, employ longitudinal designs, and incorporate validated clinical data to strengthen causal interpretation and applicability.

## Conclusion

Yet the bimodal distribution of skills shows that while a substantial subgroup navigates digital health effectively, another faces significant barriers. This heterogeneity supports the need for routine DHL screening in psychiatric care, complemented by targeted training in higher-order skills such as critical appraisal and application. Using the SIMHL framework, our findings indicate that DHL is shaped by structural factors (e.g., employment, gender) and digital engagement (e.g., use of health-related websites), rather than illness severity. Thus, study designs and policies should explicitly consider these determinants when developing digital tools for BD. Ultimately, improving DHL is not an end in itself but a strategy to ensure that individuals with BD and other SMI can fully benefit from digital innovations, thereby strengthening participation, autonomy, and wellbeing in a connected society.

## Data Availability

The raw data supporting the conclusions of this article will be made available by the authors, without undue reservation.
